# High-precision plant height measurement by drone with RTK-GNSS and single camera for real-time processing

**DOI:** 10.1038/s41598-023-32167-6

**Published:** 2023-04-18

**Authors:** Yuta Matsuura, Zhang Heming, Kousuke Nakao, Chang Qiong, Iman Firmansyah, Shin Kawai, Yoshiki Yamaguchi, Tsutomu Maruyama, Hisayoshi Hayashi, Hajime Nobuhara

**Affiliations:** 1 Department of Intelligent and Mechanical Interaction Systems, Graduate School of Science and Technology, University of Tsukuba, Ibaraki, 305-8573 Japan; 2 School of Computing, Tokyo Institute of Technology, Meguro City, Tokyo, Japan; 3 Faculty of Engineering, Information and Systems, University of Tsukuba, Ibaraki, Japan; 4 Faculty of Life and Environmental Sciences, University of Tsukuba, Ibaraki, Japan

**Keywords:** Computational biology and bioinformatics, Engineering, Optics and photonics

## Abstract

Conventional crop height measurements performed using aerial drone images require 3D reconstruction results of several aerial images obtained through structure from motion. Therefore, they require extensive computation time and their measurement accuracy is not high; if the 3D reconstruction result fails, several aerial photos must be captured again. To overcome these challenges, this study proposes a high-precision measurement method that uses a drone equipped with a monocular camera and real-time kinematic global navigation satellite system (RTK-GNSS) for real-time processing. This method performs high-precision stereo matching based on long-baseline lengths (approximately 1 m) during the flight by linking the RTK-GNSS and aerial image capture points. As the baseline length of a typical stereo camera is fixed, once the camera is calibrated on the ground, it does not need to be calibrated again during the flight. However, the proposed system requires quick calibration in flight because the baseline length is not fixed. A new calibration method that is based on zero-mean normalized cross-correlation and two stages least square method, is proposed to further improve the accuracy and stereo matching speed. The proposed method was compared with two conventional methods in natural world environments. It was observed that error rates reduced by 62.2% and 69.4%, for flight altitudes between 10 and 20 m respectively. Moreover, a depth resolution of 1.6 mm and reduction of 44.4% and 63.0% in the error rates were achieved at an altitude of 4.1 m, and the execution time was 88 ms for images with a size of 5472 × 3468 pixels, which is sufficiently fast for real-time measurement.

## Introduction

By 2050, the current crop yields will be insufficient to feed the world population, and more stable crop production must be achieved against a backdrop of climate stresses that limit yields owing to pests and pathogens, precipitation, heat waves, and other weather extremes. Eliminating hunger is an important global goal and is included in sustainable development goals^1,2^. Drones are used to boost agricultural productivity by spraying pesticides, fertilizers, and collecting data^3–5^. In plant breeding, there is a demand for high-throughput processing that can measure several plants in a vast field with high precision, and the use of drones is beginning to produce promising results^6,7^. In plant breeding, more precise measurements than those being currently achieved on a large scale can improve breeding efficiency and productivity. Specifically, it is essential to measure the height of each crop with high accuracy, and it would be innovative in the world of breeding if this measurement can be achieved in millimeters.

The use of drones in plant phenotyping experiments has been extensively studied^8,9^. Conventional measurement methods for such purposes are mainly based on structure from motion (SfM) based method and they have three primary challenges. The first is that they require extensive computation times because a large number of aerial images (several hundred) must be processed. The second is that the measurement accuracy is not high, that is, the measurement resolution is in centimeters. The third is that the cost of redoing a failed 3D reconstruction is very high. Specifically, if the 3D reconstruction result fails, a large number of aerial photos must be captured again.

The question of this study is: “Is it possible to realize millimeter-accurate height measurements in real-time using a drone and monocular camera?”.

**Figure 1:**
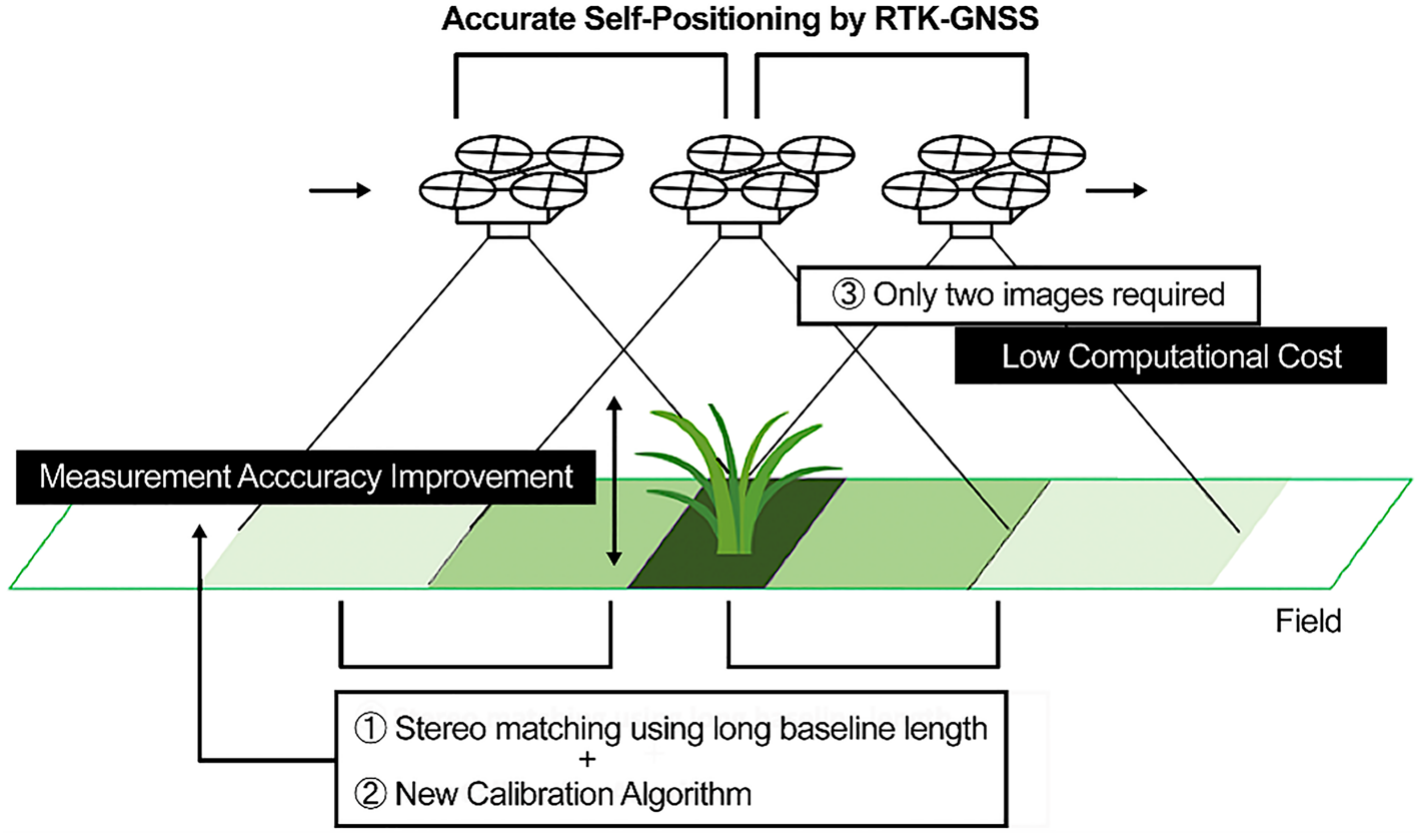
Overview of the proposed method.

To overcome the challenges of conventional methods (that is, to answer the question of this study) and realize plant height measurement with millimeter resolution in a real field, a stereo matching method is proposed that uses a drone equipped with a real-time kinematic global navigation satellite system (RTK-GNSS) and a single high-resolution monocular camera, as shown in Fig. 1. The novelty of the proposed method is that it performs high-precision stereo matching based on long baseline lengths (1.45 m and 0.81 m, Experiments 1 and 2, respectively) during the flight by linking the RTK-GNSS and aerial image capture points^10,11^. The proposed method can estimate crop height using only two images (stereo matching), thereby dramatically reducing the computation time and enabling real-time processing. Thus, if the height measurement fails, it can be re-measured by immediately capturing two more images. By enlarging this length according to the altitude of the drone, sufficient parallax can be maintained at any altitude to achieve precise stereo matching. The flexibility of the proposed system enables plant height measurements at a high resolution in a real field. However, the angle of the camera relative to the ground changes during flight, and precise calibration is required for every image pair. Because the baseline length of a standard stereo camera is fixed, once calibrated on the ground, the camera does not need to be calibrated again during the flight. However, because the baseline length is not fixed, the suggested approach necessitates fast calibration in flight. Therefore, a new calibration algorithm is proposed based on the zero-mean normalized cross-correlation (ZNCC) and two stages least square method. ZNCC is a simple algorithm, in addition to this, a two-step regression analysis based on the least-squares method can be used to achieve robust and fast processing. The photography and GNSS positioning were controlled through a microcomputer, and the plant height was calculated using the difference between the plant and ground depths obtained through stereo matching.

To verify the effectiveness of the proposed method, two experiments were performed under different conditions in a real-world environment. In the first experiment, images of fake plants were captured under a wide range of conditions in a real field environment from altitudes between 10 and 20 m, and plant heights were measured using the proposed method and well-known state-of-the-art (SOTA) conventional SfM-based methods^12–19^, Pix4Dmapper and Metashape. In the second experiment, a comparison between the methods was performed in a buckwheat field for a more realistic environment. The images were captured from an altitude of 4.1 m, and the proposed and SfM-based methods were applied to them. The true value is the actual height of the field, measured by us by hand, and the errors are calculated using these true values and the estimated value by the proposed method and conventional methods (Pix4D and Metashape).

From the results of the first experiment, it was evident that the proposed method realized a higher depth resolution of 16 mm in and reduced the error rates by 62.2% and 69.4%, where the reduction ratio corresponds to the difference between the error of the proposed method and the error of the conventional method divided by the error of the conventional method (Pix4Dmapper and Metashape respectively), at flight altitudes between 10 and 20 m. Moreover, in the second experiment, a depth resolution of 1.6 mm and a reduction in errors of 44.4% and 63.0% were achieved for an altitude of 4.1 m. Moreover, the execution time of the proposed method on a NVIDIA GTX 1080 TI GPU was 88 ms for images with a size of 5472 × 3468 pixels, which is sufficiently fast for real-time measurement and can suggest if a re-measurement is required, which can be done immediately by capturing two more images.

The remainder of this paper is organized as follows. Related work are described in “Related works”, and the proposed method is described in detail in “Experimental evaluation”. In “Discussion”, the effectiveness of the proposed system is verified through two experiments conducted in a real environment. “Methods” discusses the experimental results.

## Related works

### Limitations of drone measurements in the field

In recent years, precision agriculture has been promoted by the introduction of drones^20,21^. The advantage of drone measurements is that enormous amounts of field data can be obtained in a short time^22^. Additionally, drones allow low-altitude photography of a field at a very low cost, which is impossible through satellites and manned aircraft. Two methods are often used for measurement: photographing fields using a camera mounted on a drone and laser scanning from a drone. Related studies have been conducted to measure plant height using shooting measurements in combination with RTK-GNSS through drones or laser scanning by drones, and the root-mean-square errors obtained were 110 and 80 mm, respectively^23^. Therefore, previous studies are still limited to measurements with centimeter resolution and have not yet achieved millimeter resolution, and accurate measurements are obtained manually. If drones can achieve millimeter resolution, they can completely replace manual measurements and have a significant impact on precision agriculture.

### Limitations of SfM (general context)

SfM is a widely used conventional measurement method that uses images captured by a drone. It is a 3D measurement method that uses the principle of stereo photography and develops a 3D reconstruction from various directions based on features extracted from several image groups with a high overlap ratio and matching them^24^. The use of drones and SfM has been successful in various applications, including agricultural measurements, urban mapping, and river monitoring. Also accurate location estimation using RTK-GNSS has been suggested to be effective^25,26^. A 3D reconstruction study on the coast using drones and SfM achieved a depth resolution of 50 mm^27^. A root-mean-square error of 46.05 mm in the vertical direction was also reported in topographic mapping performed by drone and SfM^28^. The advantage of SfM is that it can easily generate a 3D model from the image group using the camera position. But the point group data obtained by SfM are not sufficiently accurate and it is difficult to measure the plant height with high precision, such as in millimeters. Another problem is that several image sets with a high overlap ratio photographed from multiple directions are generally required for 3D reconstruction, which means that the computational time of SfM is significant. In this study, the well-known and SOTA conventional SfM methods, Pix4Dmapper and Metashape, are considered^29^.

### Limitations of SfM (agricultural context)

The primary purpose of breeding is to improve the growth of a target crop in a particular environment, and increasing yields and improving drought and flood resistance could also be considered. In the breeding field, the process of evaluating the performance of genotypes for a particular set of traits in a particular environment is called plant phenotyping. In the past, plant phenotyping was labor-intensive, but automated and high-throughput plant phenotyping platforms based on large-scale aerial remote sensing are now available^12,13^. Large-scale plant phenotyping systems using unmanned aerial vehicles (UAVs) have also been proposed, and it has been suggested that the use of drones is effective in reducing the enormous workload required for plant phenotyping^14,15^. Plant height data are an important parameter for evaluating growth, and drones are attracting attention as a useful technology for measuring them. Remote sensing with a fixed camera enables high-precision measurement of plant height^16,17^; however, the accuracy of measurement decreases when the position and attitude of the camera, such as drone measurement, need to be specified. It was reported that the error in the plant height measured using drones is 88.39 mm^18^ or approximately 100 mm^19^, and further improvement in accuracy is required.

**Figure 2:**
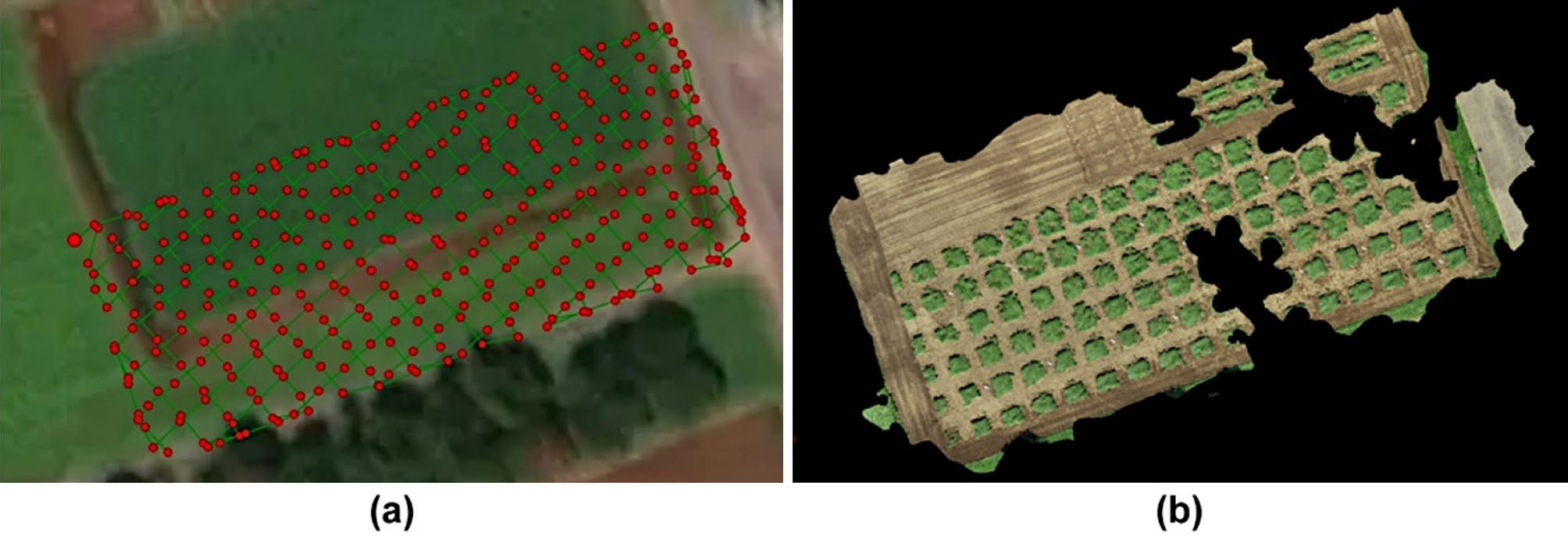
(**a**) Flight route of Phantom 4 RTK and (**b**) 3D reconstruction result by Pix4Dmapper for 332 field images and their GNSS data obtained by Phantom 4 RTK.

The Pix4Dmapper is a widely used tool for SfM, and the Phantom 4 RTK is a drone system used for capturing images with GNSS data. In Phantom 4 RTK, photography and RTK-GNSS positioning are synchronized. Figure 2a shows the flight route of the Phantom 4 RTK in a test field, and the result obtained by Pix4Dmapper for 332 images and their GNSS data captured by the Phantom 4 RTK at an altitude of 8 m and an overlap ratio of 90%. Although it consumes considerable computational resources, the height accuracy achieved by the Pix4Dmapper and Phantom 4 RTK is not sufficiently accurate. As shown in Fig. 2b, there exist several areas that it fails to reconstruct in 3D. In the case of SfM, the cost of redoing a failed 3D reconstruction is very high; specifically, if the 3D reconstruction result fails, a large number of aerial photos must be captured again by the drone.

## Experimental evaluation

### Experimental setting

**Figure 3:**
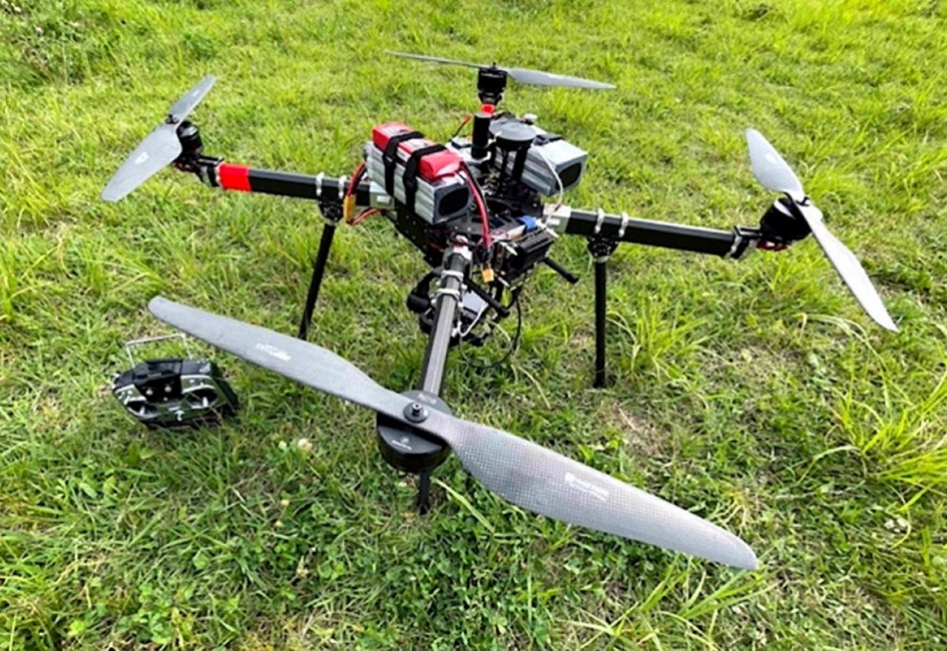
An image of the drone used.

**Figure 4:**
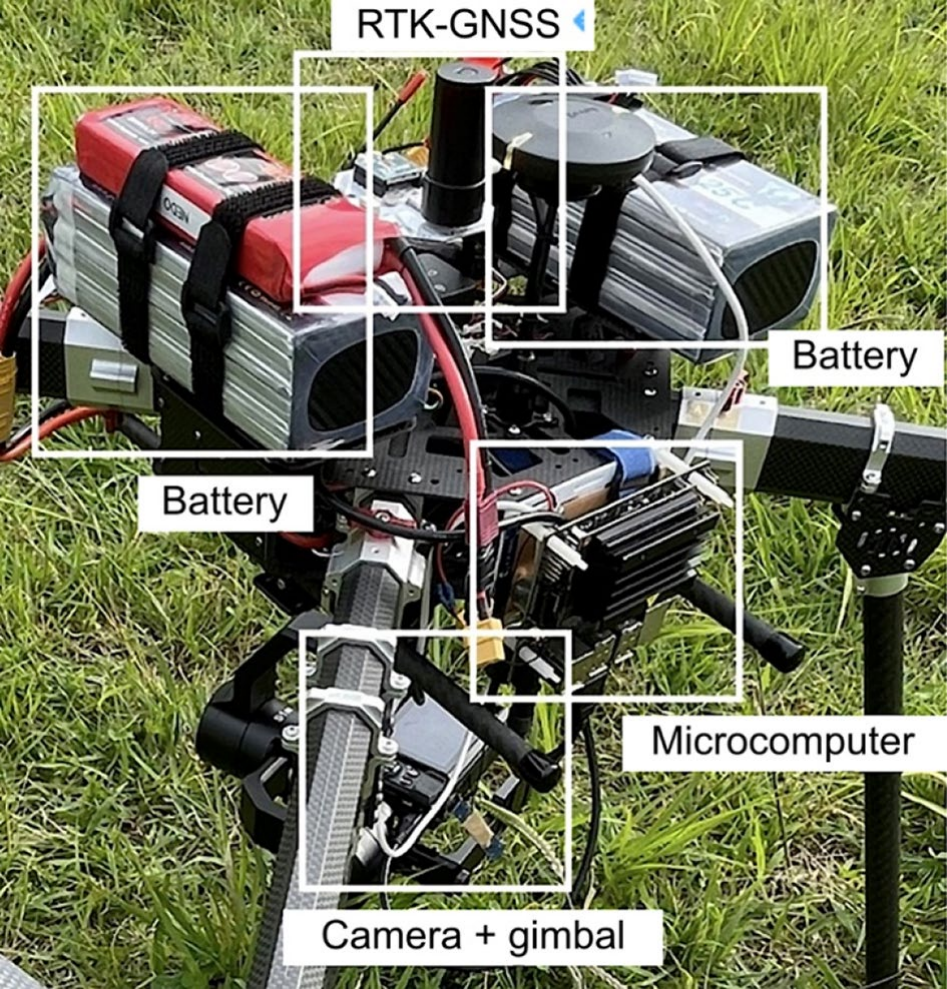
Drone equipment.

A drone equipped with the proposed system was flown under two different conditions to verify the effectiveness of the system. The plant heights obtained by the proposed system compared with those obtained by conventional SfM methods, such as Pix4Dmapper and Metashape. Figure 3 shows an image of the drone used in the evaluation. The basic configuration of the drone is shown in Fig. 4 and its specifications are listed in Table 1.

**Table 1:** Drone specifications.

Drone body	GD-X8 V2 (1000 mm)
Motors	T-motor P60 × 4
Flight controller	CUAV X7
GNSS	RTK-GNSS: ZED-F9P module
Gimbal	GREMSY S1V3
Camera	Canon PowerShot G7 X Mark 2
Micro-computer	JETSON Nano

The camera and RTK-GNSS were controlled by a microcomputer and synchronized when images were captured. The images and their positions could be monitored from the ground base via wireless communication.

In the first experiment, the height of the fake plants in the field was measured from an altitude between 10–20 m. In the second experiment, the height of buckwheat cultivation in a real field was measured from an altitude of 4.1 m.

### First experiment

**Figure 5:**
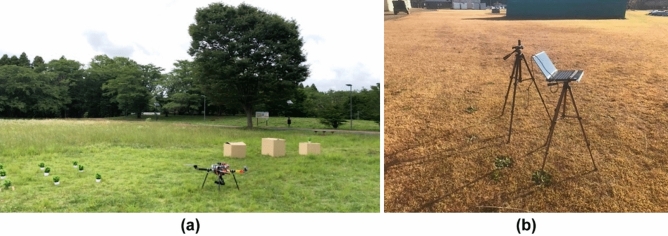
Environment of first experiment: (**a**) test field and (**b**) base station.

To evaluate the performance of the proposed method, a fake plant and three boxes were placed on the ground, and one box had a fake plant in it. Their images were captured using a drone on the field. The captured images were used to perform 3D reconstruction through the proposed and conventional methods (Pix4Dmapper and Metashape), and the obtained plant height accuracy was evaluated. Figure 5a shows the field used for this experiment and Fig. 5b shows the base station used to communicate with the drone. Table 2 shows a sequence of the latitudes (degree) and longitudes (degree) obtained by RTK-GNSS and linked to the images captured during flight. The number of the photograph sequence starts from 1, and it is evident that the latitudes and longitudes change as this number changes. Table 3 lists the distances between each position calculated using Eq. (4).

**Table 2:** Examples of a shooting a sequence and positions.

Shooting sequence	Latitude (°)	Longitude (°)
1	36.11878417	140.0937983
2	36.11878317	140.0937943
3	36.11878233	140.0937910
4	36.11878133	140.0937872
5	36.11878050	140.0937835
6	36.11877983	140.0937805
7	36.11877867	140.0937767

**Table 3:** Baseline lengths calculated from shooting positions (meters).

	1	2	3	4	5	6	7
1	0						
2	0.3761	0					
3	0.6895	0.3134	0				
4	1.0513	0.6752	0.3618	0			
5	1.3934	1.0173	0.7038	0.3421	0		
6	1.6727	1.2967	0.9833	0.6216	0.2795	0	
7	2.0400	1.6639	1.3505	0.9887	0.6468	0.3680	0

**Figure 6:**
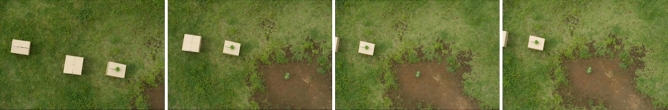
Example of a series of images.

Figure 6 shows an example of a series of images of 5472 × 3648 pixels captured from a height of 18.7 m above the ground.

**Figure 7:**
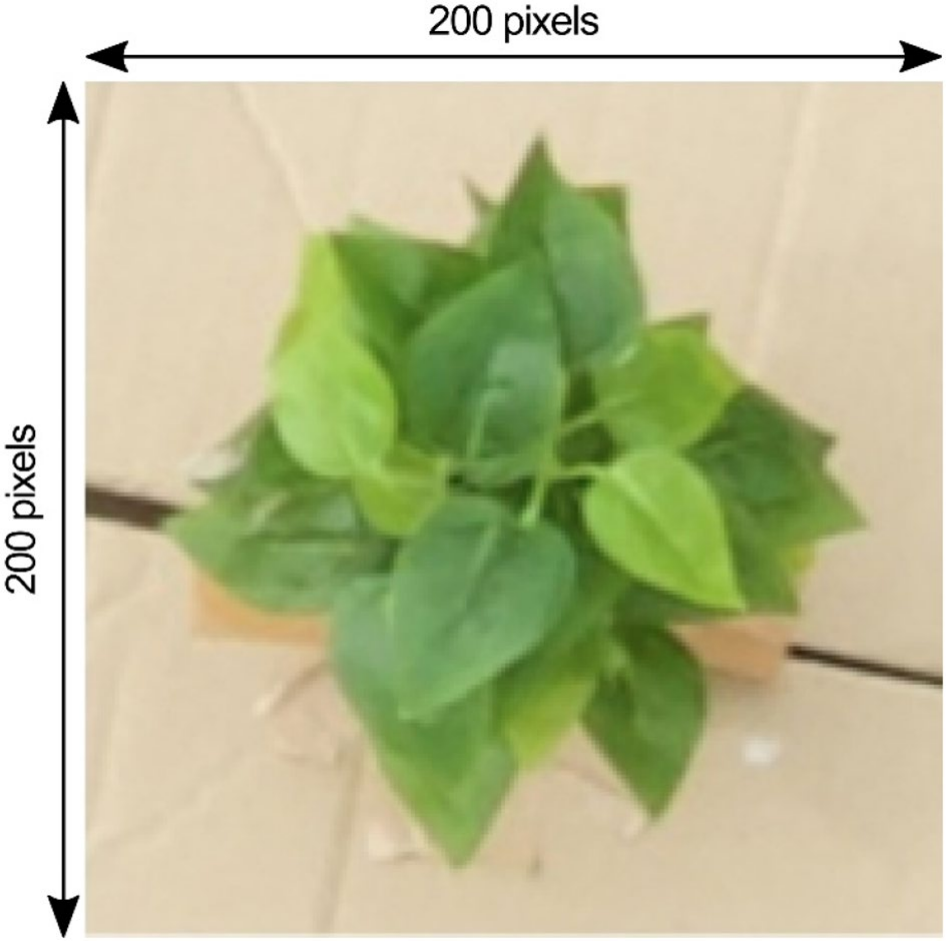
Enlarged view of the fake plant in the box.

**Figure 8:**
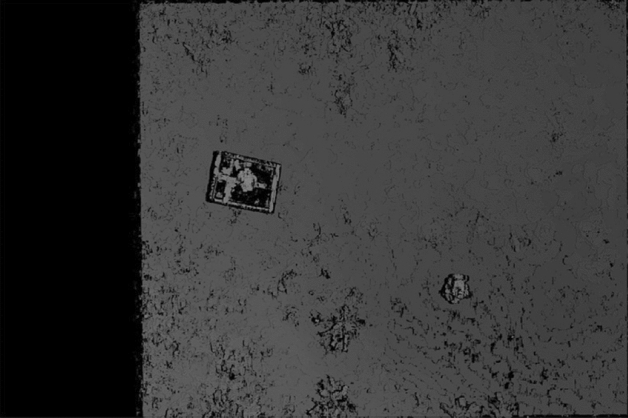
Disparity map obtained from two images in Fig. 6.

Figure 7 shows an enlarged view of the fake plant placed in the box. This size of this enlarged part is 200 × 200 pixels owing to the very high resolution of the camera used. Figure 8 shows the disparity map obtained from the two images, where each shooting position (longitude, latitude) = (36.11417632, 140.0992424), and (36.11417558, 140.0992251), and by using Eq. (4), we calculated the baseline length = 1.45 m. Given a pair of stereo images, to compute the disparity map, match all pixels in the left image with the corresponding pixels in the right image, as the first step. Then, the distance is calculated for each pair of matched pixels, as the second step. Finally, the distance values are expressed as an intensity image to obtain a disparity map. The resolution of the intensity of the image representing this disparity map corresponds to the depth resolution. In the disparity map (Fig. 8), the intensity of the highest part of the plant is 1224, and that for the lowest ground is 1188. The difference between the two values is 37, and the height of the plant measured manually on the ground is 595 mm. This means that the difference of one pixel in the disparity map translates to a difference of 16 mm in height. Thus, estimating plant heights from disparity maps is possible with a resolution of 16 mm. Although the downwash effect increases when the altitude of the drone is lowered, the disparity maps can be obtained at a finer resolution.

**Figure 9:**
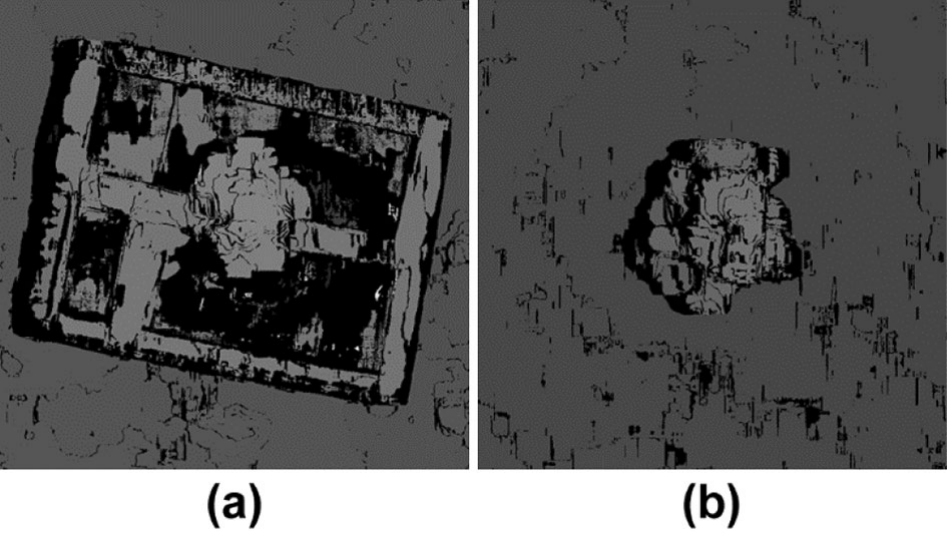
Two enlarged parts of the disparity map shown in Fig. 8. (**a**) Plant in the carboard box and (**b**) plan on the ground.

Figure 9 shows two enlarged parts of the disparity map: the cardboard box containing the fake plant and the fake plant placed on the ground. As shown in Fig. 9, stereo matching was successful for the plants. The black pixels in the images indicate stereo matching failures. The failures on the plants are primarily caused by occlusion, whereas on the box they are caused by its flat surface that has minor changes. The true value is the actual height of the field, measured by us by hand. In Experiment 1, the two false plant heights are shown in Fig. 9a,b, and the true values are 24.0 cm and 59.5 cm, respectively. The maximum resolution of the stereo vision in this experiment was 16 mm as described above. However, the height of the plant estimated by the system was 561 mm, which indicates an error of 34 mm. Stereo matching was implemented on an NVIDIA GTX 1080 TI GPU, and it was confirmed that one disparity map could be calculated in 88 ms from two images with a size of 5472 × 3648 pixels when the range of the disparity from the minimum to the maximum was 128.

**Figure 10:**
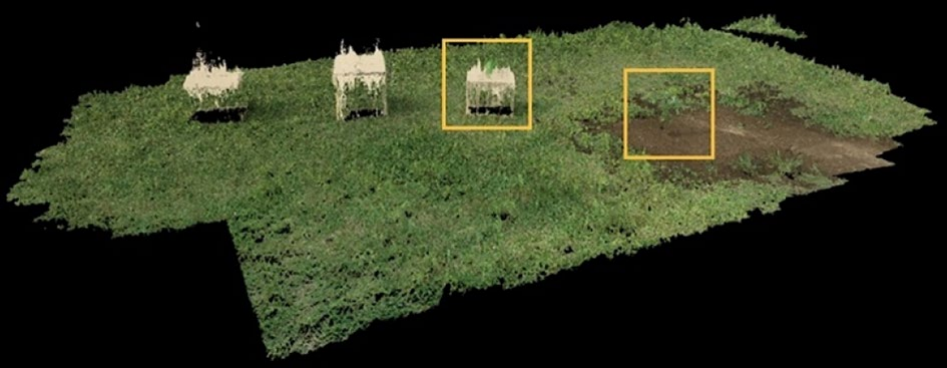
3D reconstruction through Pix4Dmapper.

**Figure 11:**
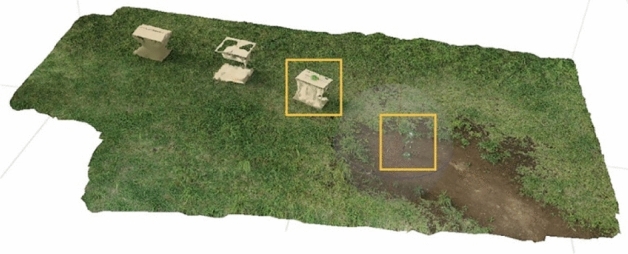
3D reconstruction through Metashape.

To compare the proposed method with the conventional methods, a 3D reconstruction was performed by providing a series of images to Pix4Dmapper and Metashape. Figures 10 and 11 show the 3D reconstructions obtained through Pix4Dmapper and Metashape, respectively, using 104 images and their GNSS data captured by the drone equipped with RTK-GNSS and a single monocular camera. In Figs. 10 and 11, the plant details are not reconstructed, and it is difficult to measure the height with high precision, which was achieved using the proposed method. Table 4 lists errors of the proposed method and the two conventional methods for plant height measurements. The proposed method is superior in that the height can be obtained using only two images, whereas 3D reconstruction methods require 104 images. This enables the images to be captured again during the same flight in the case of measurement failures. Regarding the measurement accuracy, Pix4Dmapper and Metashape had errors of 90 mm and 111 mm, respectively, from the true value, and the error using the proposed method was 34 mm.

**Table 4:** Errors of the proposed method and the two conventional methods for the first experiment.

	Proposed method	Pix4Dmapper	Metashape
Error (cm)	3.4	9.0	11.1
Number of images	2	104	104

### Second experiment

**Figure 12:**
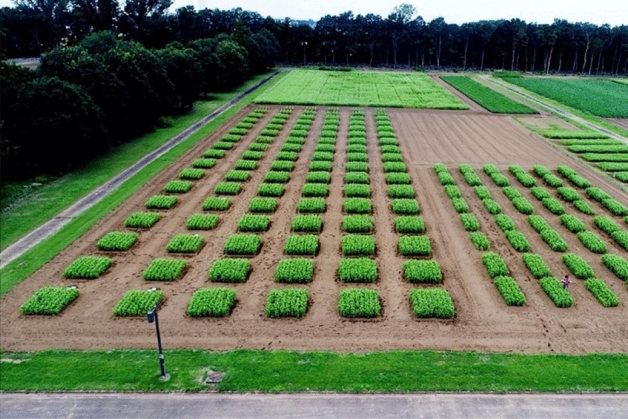
Target field of the second experiment.

As previously mentioned, in the agricultural context, it was reported that the error of the plant height measured using drones is 88.39 mm^18^ or approximately 100 mm^19^, and further improvement is required. The first experiment confirmed that high-precision growth measurement of plants was possible using the proposed method. In this section, the results were obtained for a real field under cultivation, that is, for the agricultural context. For this experiment, buckwheat was grown in a test field, as shown in Fig. 12. It is 75 m long and 30 m wide, and contains approximately 50,000 buckwheat plants.

**Figure 13:**
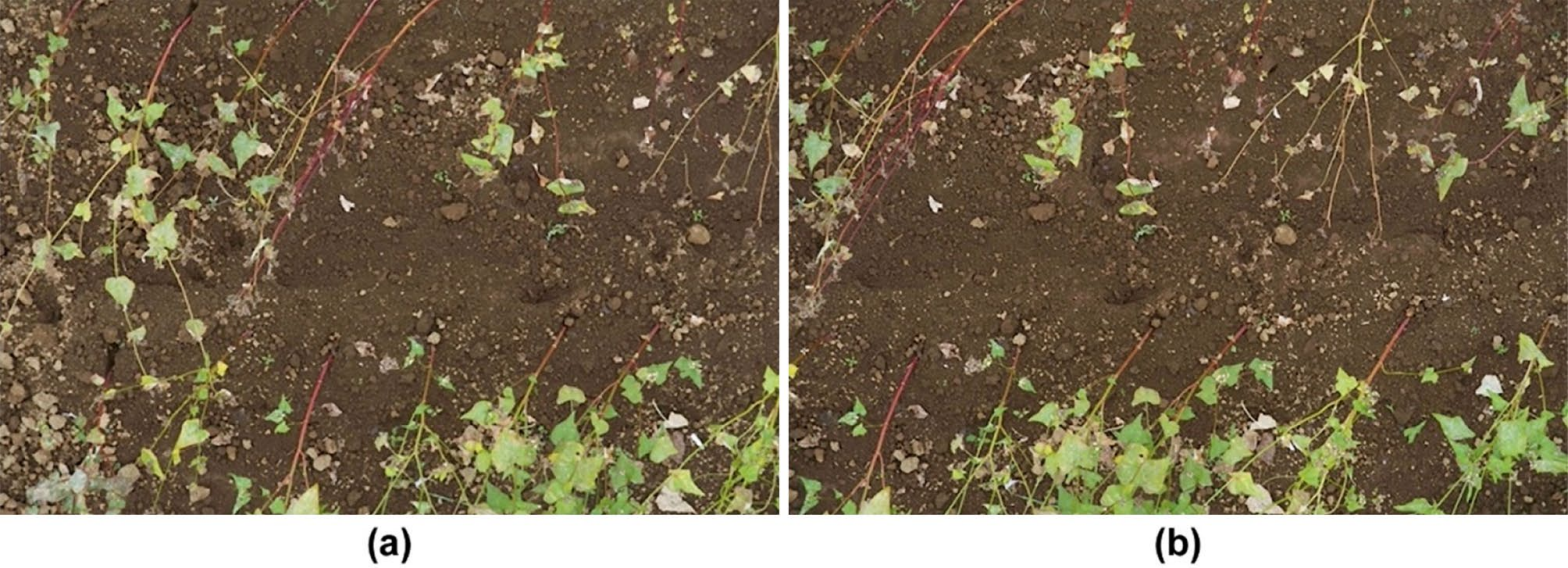
Two images of buckwheat in the test field.

**Figure 14:**
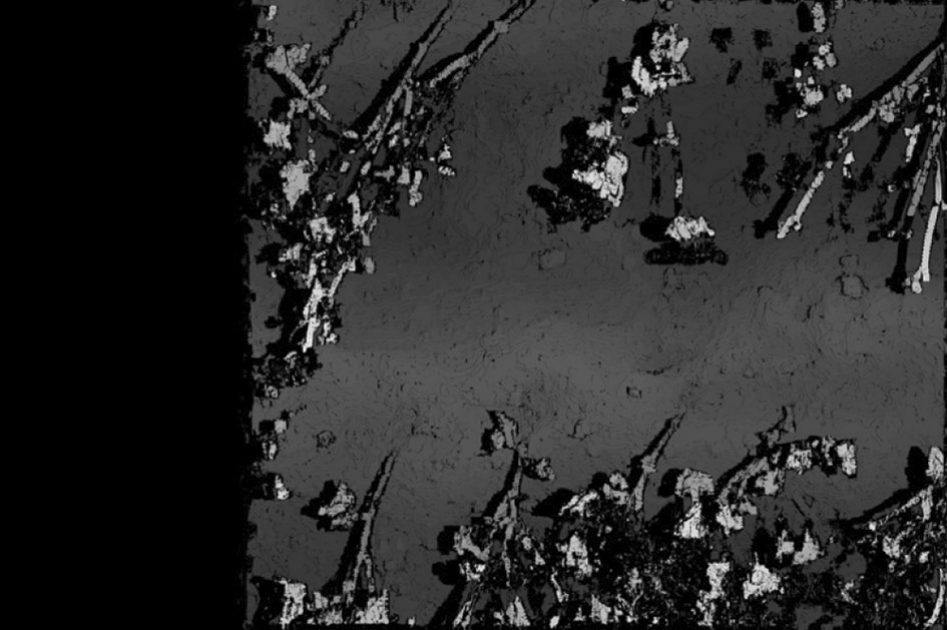
Disparity map obtained from the two images shown in Fig. 13.

The same process as in the first experiment was performed in this experiment. Figure 13a,b shows two images captured from a height of 4.1 m, which is closer than in the first experiment, and we obtained a baseline length of 0.81 m using Eq. (4) and the shooting positions. Figure 14 shows the disparity map obtained from the two images in Fig. 13. In the disparity map, black pixels indicate failures of stereo matching, which are mainly caused by occlusion. The true value is the height of one of the buckwheat plants selected within the measurement range and measured by hand, as shown in Fig. 14. The maximum disparity was for the highest buckwheat plant at 1573 pixels, and the minimum disparity for the ground was at 1374 pixels. Thus, the difference was 199 pixels and the manually measured plant height was 570 mm. This indicates a change of 2.9 mm in height when the disparity differs by one pixel. Therefore, it was possible to estimate the height of plants from the disparity map with a resolution of 2.9 mm. This indicates that plant height measurement with millimeter accuracy, which is the goal of the proposed method, can be achieved in a real field.

**Figure 15:**
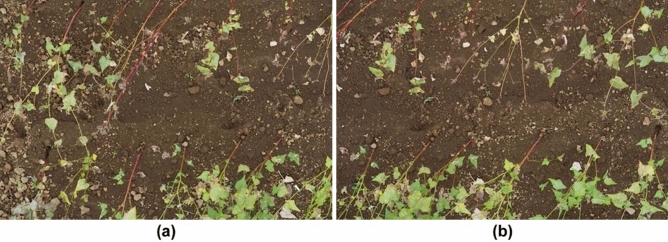
Two images with longer baseline lengths than those shown in Fig. 13.

**Figure 16:**
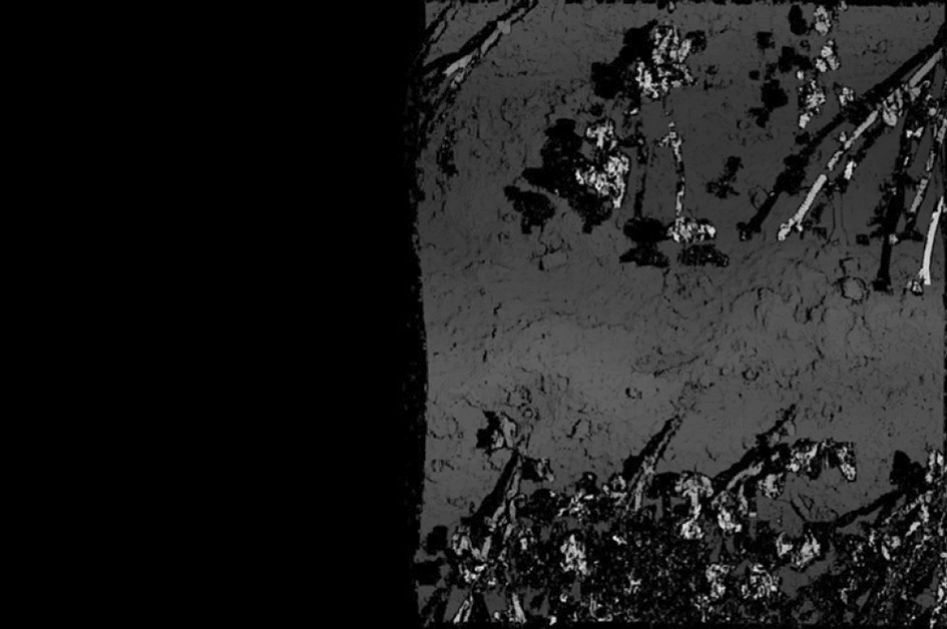
Disparity map obtained from the two images shown in Fig. 15.

A higher stereo matching resolution can be obtained by enlarging the baseline length, that is, the distance between the shooting locations of the two images. Figure 15a,b shows two images with a longer baseline, and Fig. 16 shows the disparity map obtained from them. In this disparity map, the maximum disparity (number of levels) is 2,320 pixels, whereas the minimum is 1,957 pixels. Thus, the difference is 363 pixels, and the manually measured height of the highest buckwheat in the images was 570 mm. Thus, it is possible to estimate the height of a plant from the disparity map with a resolution of 1.6 mm. By enlarging the baseline, the overlap ratio of the two images decreases, and accurate calibration and stereo matching become challenging. The overlap ratio of the two images in Fig. 15 is slightly larger than half of the image width. This overlap ratio can be easily determined from Fig. 16. If the overlap ratio is more than half of the image width, the two images can be calibrated using the proposed calibration algorithm. To compare with the proposed method, 84 images and their GNSS data were captured by a drone equipped with RTK-GNSS and a single high-resolution camera and this data was provided to Pix4Dmapper and Metashape for 3D reconstruction. Table 5 lists the errors obtained by the proposed method and two conventional methods for plant height measurements. In the proposed method, the accuracy of the height detection was evaluated using eight image pairs with different baseline lengths. The results are presented as the average and standard deviations. The most accurate measurement had an error of 9 mm, and the proposed method has a smaller error in plant height measurements compared to Pix4Dmapper and Metashape. Additionally, there were several areas in their reconstruction where point groups could not be obtained because even 84 input images were insufficient, A greater number of images is required to obtain the complete 3D reconstructions of the experimental field, but this number can be discovered at the ground base only after the shooting flight.

**Table 5:** Comparison between the proposed method and the two conventional methods in the second experiment.

	Proposed method	Pix4Dmapper	Metashape
Error (cm)	$5.0$	9.0	13.5
Number of images	2	84	84

## Discussion

The conventional methods of measurement used in these experiments, especially plant height measurement using drones, includes structure from motion (SfM), light detection and ranging (LiDAR), and methods using binocular stereo cameras^30,31^. However, the measurement accuracy of the methods using a stereo camera mounted on a drone is insufficient because their short and fixed baseline length does not provide sufficient parallax. For example, the baseline of ZED camera^32^ is 120 mm, Kinect is 80 mm^33^, respectively. LiDAR is high accuracy; however, it has challenges owing to its weight and cost. Therefore, the most commonly used method is the combination of SfM and a large number of aerial images captured by drones. Conventional methods have challenges in terms of accuracy, weight, and cost, as listed in Table 6.

**Table 6:** Comparison between the proposed and conventional methods/devices structure from motion (SfM) and light detection and ranging (LiDAR).

	SfM	Stereo Camera	LiDAR	Proposed method
Accuracy	Bad	Bad	Average	Good
Weight	Good	Good	Bad	Good
Cost	Good	Good	Bad	Good

In the case of proposed method, the height detection resolution obtained through stereo matching was 16 mm in the first experiment and 1.6–2.9 mm in the second experiment. The accuracy of the proposed method can be obtained from the camera specifications and experimental conditions (baseline length, etc.).

This result is reasonable when we consider the ratio of the flight height, which was 18.7 m and 4.1 m for the first and second experiment, respectively, and the overlap ratios of the images shown in Figs. 8, 14 and 16. Higher resolution can be achieved for a smaller overlap ratio, that is, for a longer baseline length. Considering these results, height detection with millimeter to centimeter resolution was possible with the camera used in these experiments by changing the flying height and overlap ratio of the two images. By multiplying the altitude *k* times, the entire field can be photographed with *1/k* number of images when the overlap ratio is the same; however, the height detection accuracy also becomes approximately 1*/k* times. The flying height and overlap ratio can be determined based on these requirements.

Although the errors in the estimated plant heights in these experiments were smaller than those of conventional 3D reconstruction tools, they were still 34 and 50 mm, which is significantly more than in the height detection accuracy through stereo matching. This can be because of two reasons: first, the position accuracy of RTK-GNSS is in centimeters and not millimeters; second, the ground is not flat, and it is not easy to find the root position of the target plant. Despite these factors, it is quite possible to achieve observation resolution in the order of millimeters in the context of agriculture as the same fields are observed regularly. This specific method is presented below in the “Methods” section.

## Methods

### Overview

**Figure 17:**
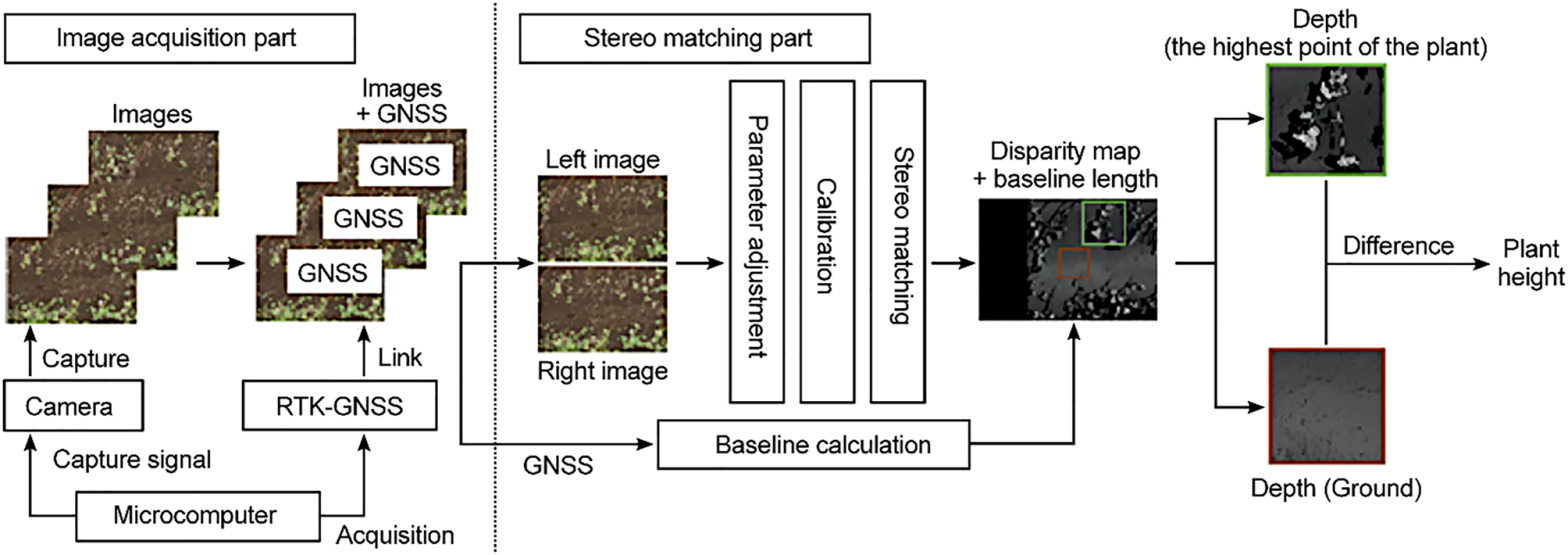
Process flow of the proposed system.

In this study, a stereo-matching method to accurately measure the crop heights using a drone equipped with a single high-resolution monocular camera and RTK-GNSS is proposed. The process flow of the proposed system is illustrated in Fig. 17.

The proposed system can be roughly divided into two parts: image acquisition with RTK-GNSS positioning and high-precision height measurement through stereo matching. For image acquisition, the camera and RTK-GNSS are controlled by a microcomputer, and photography and RTK-GNSS positioning are performed simultaneously. The acquired images and their position data are then linked and stored in memory. For stereo matching, the baseline length is first calculated from the position data of the two images used for stereo matching. The baseline length is used to reduce the computational load of calibration and calculate the distance from the disparity map to the objects in the images. The parameters used in calibration and stereo matching are adjusted for the two images. The two images are then calibrated, and a disparity map is generated by stereo matching the two calibrated images. Let *b* be the baseline length and *f* be the focal length of the camera. When the disparity of a point in the disparity map is *d*, the distance to point *l* is given by

1 $$\begin{array}{c}l=f\frac{b}{d}.\end{array}$$

Thus, the height of a plant, *p*_*h*_, is given by

2 $$\begin{array}{c}{p}_{h}=f\frac{b}{{d}_{g}}-f\frac{b}{{d}_{p}},\end{array}$$

where *d*_*p*_ is the disparity of the highest point of the plant and *d*_*g*_ is the disparity of the ground around the plant. Mounting a binocular stereo camera on a drone is simpler than using a monocular camera alone and does not require calibration during the flight. However, the baseline length of the stereo camera is restricted by its body size and is therefore short and fixed. Suppose that *b* in Eq. (1) is lengthened by *k* times. Subsequently, for a point at the same distance, *d* becomes larger by *k* times to maintain the equation equality. This implies that the disparity value of the target object can be controlled by changing the baseline length. Next, consider the difference in distance that can be detected when the disparity is changed by one. The difference is given by:

3 $$\begin{array}{c}\Delta d={l}_{d}-{l}_{d+1}=f\frac{b}{d}-f\frac{b}{d+1}=f\frac{b}{d\left(d+1\right)}=\frac{{l}_{d}}{d+1}.\end{array}$$

By reducing ∆*d*, the stereo matching detection resolution can be improved. There are two approaches to reduce ∆*d*: reducing *l*_*d*_, namely, reducing the distance between the camera and target, and increasing the baseline length, that is, increasing *d*. However, the distance to the target object cannot be sufficiently reduced, especially for plants that can be easily shaken by the drone downwash. Therefore, increasing the baseline length is the only solution to achieve a high detection accuracy, but it is impossible through stereo cameras. In the proposed system, by capturing two images from the drone, the height of the plants in the overlapped area can be obtained through stereo matching. In contrast, an enormous amount of aerial imagery is provided after the flight in traditional methods to reconstruct the 3D model of the field. The proposed system enables real-time detection of stereo matching failures and retakes the images of the region during the same flight. It is also possible to investigate the area of interest in detail when flying a drone.

### Image acquisition with RTK-GNSS positioning

In the proposed system, plant height can be accurately measured by maintaining a long baseline length by periodically capturing images of the field using a single monocular camera. This camera is attached to a drone with a gimbal to counter body shakes during flight. The shooting position of each image is given by the RTK-GNSS, which is a self-positioning system capable of measuring its position with an error of a few centimeters. The camera and RTK-GNSS are controlled by a microprocessor attached to the drone, and the photography and RTK-GNSS positioning are synchronized. The drone is connected to a computer on the ground via wireless communication, and the captured images and their positions can be checked in real time. In addition to the position data linked to the images, RTK-GNSS positioning is performed at intervals of 0.1 s. This precise tracking of drone flight guarantees the correctness of the GNSS data linked to each captured image. To achieve high calibration and stereo matching accuracy, the overlap ratio of the two images must be greater than half of the image width. This requirement can easily be satisfied through precise tracking.

The distance between the two shooting points, points 1 and 2, can be calculated using spherical trigonometry; this distance is used as the baseline length. The parameters are defined as follows:

4 $$\begin{array}{l}GNSS\, Data\left\{\begin{array}{l}\begin{array}{ll}r& \mathrm{Earth \,radius},\end{array}\\ \begin{array}{ll}La1& \mathrm{Latitude\, of\, point\, }1,\end{array}\\ \begin{array}{l}\begin{array}{ll}Lo1& \mathrm{Longitude\, of\, point\, }1,\end{array}\\ \begin{array}{ll}La2& \mathrm{Latitude \,of \,point \,}2,\end{array}\\ \begin{array}{l}\begin{array}{ll}Lo2& \mathrm{Longitude\, of\, point\, }2,\end{array}\\ \begin{array}{ll}{L}_{a3}=\frac{{L}_{a1}-{L}_{a2}}{2}& \end{array}\\ \begin{array}{cc}{L}_{o3}=\frac{{L}_{o1}-{L}_{o2}}{2}& \end{array}\end{array}\end{array}\end{array}\right.\end{array}$$

Then, the baseline length *b* is obtained as:

5 $$\begin{array}{c}b = 2r \,arccos\sqrt{{\mathrm{sin}}^{2}\left({L}_{a3}\right)+\mathrm{cos}\left({L}_{a1}\right)\mathrm{cos}\left({L}_{a2}\right){\mathrm{sin}}^{2}\left({L}_{o3}\right)}\end{array}$$

### High-precision stereo matching

Because of the gimbal attached to the camera, the change in the camera direction during a short-distance flight for capturing two images is not very large; however, precise calibration of the two images is necessary to realize accurate stereo matching. Several algorithms have been proposed for calibration and stereo matching. In (vision.middlebury.edu), datasets for stereo vision that may or may not require calibration are given, and many algorithms have been competing to achieve the best performance on them. In the proposed system, a new calibration algorithm and a well-known stereo-matching algorithm, called zero-mean normalized cross-correlation (ZNCC), are used. In most calibration algorithms, the matching of feature points in the left and right images is used to detect the rotation and movement of the right image. In the proposed algorithm, the matching of lines, and not each feature point, is used. This is because several matching points can easily be discovered through ZNCC by using small soil blocks on the ground. In the proposed approach, the matching errors are eliminated in the following two stages:

- by using least-squares straight lines calculated from the matching points, and
- using multiple least-squares straight lines.

In both stages, strong constraints must be satisfied for the matching points and lines, and those with questionable accuracy can be easily eliminated.

The accuracy of ZNCC is not very high for stereo vision benchmark sets, mainly owing to artificial objects that often do not have sufficient changes on their surfaces. For plants and the ground that have sufficient changes on their surfaces, this algorithm has a good performance, as shown in the next section. Another reason for using ZNCC is that it can be efficiently accelerated by using GPUs^34^.

The proposed calibration algorithm consists of two phases: (a) a rough rotation around the z-axis, which is the axis vertical to the ground, and scaling based on the corner detection in^35^; and (b) precise detection of the three rotation angles and height movement during the flight, and the camera tilt when the left image is captured. Calibration is performed by assuming that the ground is a flat plane.

In the first phase, the obvious corners on the ground are searched for in the two images, and their matching is performed using ZNCC. By limiting the candidates using the position information provided by RTK-GNSS. the two images are rotated using the matching results, and the right image is scaled such that its horizontal lines are parallel to the moving direction of the drone.

In the second phase, the following steps are performed:

(i) For the ground points on the horizontal line *y* = *C*_*i*_ in the left image, the matching points are searched using ZNCC in the region *y* = *C*_*i*_ ± *S*_*R*_ in the right image. *S*_*R*_ is an empirically determined small constant. This region is further reduced using the matching results from the first phase. Using the low-error matching points, the least squares straight line *y* = *a*_*i*_*x* + *b*_*i*_ that corresponds to *y* = *C*_*i*_ is obtained. Let the line pair be (*L*_*i*_ : *y* = *C*_*i*_*, R*_*i*_ : *y* = *a*_*i*_*x* + *b*_*i*_).
(ii) Assuming that the left image is parallel to the ground, which indicates no camera tilt, the three rotation angles *β*, *γ*, and *α* of the right image around the *y, z,* and *x* axes, respectively, against the left image are calculated as follows, and *R*_*i*_ and the coordinates of the matching points on them are rotated step-by-step: a) *β* is calculated such that the gradients of all *Ri* become equal
  6 $$\begin{array}{c}\beta ={\mathrm{tan}}^{-1}\left(f\frac{{a}_{i}-{a}_{j}}{{b}_{i}-{b}_{j}}\right),\end{array}$$
  where *f* is the focal length of the lens,b) *γ* is calculated such that all *R*_*i*_ become parallel to *x*-axis (*γ* = tan^*−*1^(*a*_*i*_)),c) *α* is calculated such that all distances between *R*_*i*_ become the same:
  7 $$\begin{array}{c}\alpha ={\mathrm{tan}}^{-1}\left(-\frac{f\left({b}_{i}+{b}_{k}-2{b}_{j}\right)}{{b}_{i}{b}_{j}+{b}_{j}{b}_{k}+{b}_{k}{b}_{i}}\right)\end{array}$$
(iii) The movement along the *y*-axis of the right image, *∆y*, is calculated such that the weight balance of *b*_*i*_ becomes the same as that of *C*_*i*_, and *R*_*i*_ and the coordinates of the matching points are shifted by − ∆y. The movement along the *z* axis, *∆z*, is then calculated such that *bi* becomes equal to *Ci*, and the coordinates are scaled up or down using *∆z*.
(iv) The distances to all matching points are calculated at this point based on their disparities, and the least-squares plane on which all matching points are expected to exist is also calculated. Then, assuming that each matching point *p* is on this plane, *∆py* and *∆pz* are recalculated, and *∆y* and *∆z* are recalculated such that the sum of their squared errors can be minimized. The rotated coordinates of the matching points in the right image in step (ii) are moved and scaled using the new *∆y* and *∆z*.
(v) If the left image is completely parallel to the ground, the calibration can be completed here, but in actuality it is not. Therefore, not all *R*_*i*_ become parallel, and their distances do not become equal through the above steps. This is caused by overfitting owing to the rotations by *α, β,* and* γ* angles.
(vi) For the overfitted result, which is the output of step (iv), *α′, β′,* and *γ′* are calculated such that all *Ri* become parallel, and their distances become equal after step (ii). Parameters *α′, β′,* and *γ′* can be considered as the amount of overfitting.
(vii) The original coordinates of the matching points in the right image are rotated by *β* = *β − β′*, *γ* = *γ − γ′*, and *α* = *α − α′*, and *a*_*i*_ and *b*_*i*_ of *R*_*i*_ are recalculated. At this point, *R*_*i*_ is not parallel.
(viii) Steps (iii) and (iv) are repeated. If all matching points exist on the same flat plane, and *α′, β′,* and *γ′* are estimated correctly, all *R*_*i*_ become parallel and their distances become equal, but this does not occur. Steps from (vi) onwards are repeated until the sum of the squared errors becomes lower than a given threshold.
(ix) Finally, using the least-squares plane *z* = *ax* + *by* + *c*, the tilt of the left image is obtained as *βb* = ${\mathrm{tan}}^{-1}(a)$ and *αb* = ${\mathrm{tan}}^{-1}\left(-b \mathrm{cos}(\beta b)\right)$*.*

Using these steps, the tilt of the left image, *βb* and *αb*, the rotation and movement during the flight to capture the right image, *β, γ, α, ∆y* and *∆x* can be obtained, and the left and right images are rotated and moved using these parameters. Step (i) is the most computationally insensitive part; however, it can be efficiently parallelized on GPUs. This calibration algorithm was specifically designed for this application. The amount of rotation between the two images must be sufficiently small to discover several matching points on the same line. This is guaranteed by the gimbal attached to the camera and ground surface with sufficient changes.

### High accuracy through periodic observations

Let *L*(*t*_1_) and *R*(*t*_1_) be the images of a certain region captured at time *t*_1_ on a certain day, and *L*(*t*_2_) and *R*(*t*_2_) be the images of the same region captured at time *t*_2_ on a different day. Two disparity maps, *dm*(*t*_1_) and *dm*(*t*_2_), can then be obtained for times *t*_1_ and *t*_2_. If the ground surface does not change significantly during *t*_1_ and *t*_2_, *dm*(*t*_2_) can be scaled such that the distance to the ground becomes equal to that of dm(*t*_1_). The scaling factor can be determined by calibrating *L*(*t*_1_) and *L*(*t*_2_). Let the calibrated *dm*(*t*_2_) be *dm*^*′*^(*t*_2_). Then, the following equation is true because the distances to the ground are equal:

8 $$\begin{array}{*{20}c} {f\frac{{b\left( {t_{1} } \right)}}{{dg\left( {t_{1} } \right)}} = f\frac{{b^{{^{\prime}\left( {t_{2} } \right)}} }}{{dg^{{^{\prime}\left( {t_{2} } \right)}} }},} \\ \end{array}$$

where *b*(*t*_1_) and *b*^*′*^(*t*_2_) are the baseline lengths, and *dg*(*t*_1_) and *dg*^*′*^(*t*_2_) are the disparities of the ground in *dm*(*t*_1_) and *dm*^*′*^(*t*_2_), respectively. The ratio of the height of the target plant at times *t*_1_ and *t*_2_, namely the growth ratio *r*, can be obtained using Eq. (2) given as follows:

9 $$\begin{array}{c}r=\frac{f\frac{{b}^{{{\prime}}\left({t}_{2}\right)}}{d{g}^{{{\prime}}\left({t}_{2}\right)}}-f\frac{{b}^{{{\prime}}\left({t}_{2}\right)}}{d{p}^{{{\prime}}\left({t}_{2}\right)}}}{f\frac{b\left({t}_{1}\right)}{dg\left({t}_{1}\right)}-f\frac{b\left({t}_{1}\right)}{dp\left({t}_{1}\right)}}=\frac{{b}^{{{\prime}}\left({t}_{2}\right)}}{b\left({t}_{1}\right)}\cdot \frac{\frac{1}{d{{g}^{{{\prime}}\left({t}_{2}\right)}}}-\frac{1}{d{{p}^{{{\prime}}\left({t}_{2}\right)}}}}{\frac{1}{dg\left({t}_{1}\right)}-\frac{1}{dg\left({t}_{1}\right)}},\end{array}$$

where *dp*(*t*_1_) and *dp*^*′*^(*t*_2_) are the disparities of the target plant in *dm*(*t*_1_) and *dm*^*′*^(*t*_2_). Using Eq. (5), this equation can be rewritten as follows:

10 $$\begin{array}{c}r=\frac{d{g}^{{{\prime}}\left({t}_{2}\right)}}{d{g}^{{{\prime}}\left({t}_{2}\right)}}\cdot \frac{\frac{1}{d{g}^{{{\prime}}\left({t}_{2}\right)}}-\frac{1}{d{p}^{{{\prime}}\left({t}_{2}\right)}}}{\frac{1}{dg\left({t}_{1}\right)}-\frac{1}{dg\left({t}_{1}\right)}}=\frac{1-\frac{d{g}^{{{\prime}}\left({t}_{2}\right)}}{d{p}^{{{\prime}}\left({t}_{2}\right)}}}{1-\frac{dg\left({t}_{1}\right)}{dp\left({t}_{1}\right)}}.\end{array}$$

This equation indicates that the growth ratio can be determined from only the disparities in *dm*(*t*_1_) and *dm*^*′*^(*t*_2_) without using the RTK-GNSS position data, although it is used to reduce the computational load of calibration. In the second experiment, *dg*(*t*_1_) and *dp*(*t*_1_) are 1957 and 2320 pixels, respectively. The photography is controlled using RTK-GNSS, and it can be expected that *dg*^*′*^(*t*_2_) and *dp*^*′*^(*t*_2_) have similar values. The sensitivity for detecting this growth ratio then becomes approximately 0.28% when the plant height is 570 mm. This ratio corresponds to a height detection resolution of 1.6 mm, as demonstrated in the second experiment. Consequently, once the height of one plant is manually measured, it is possible to continue measuring the height of all plants in the range that can be captured in the same image. This constraint on the range size can be eliminated by applying these processes to the image sequence captured during one flight. Let I_0_, I_1,_ and I_2_ be the three images in a sequence, and suppose (1) I_1_ is calibrated using I_0_, *I*1*′* is generated, and (2) the height of a plant captured in both I_0_ and *I*1*′* is manually measured. Then, the height of the plants that captured in *I*1*′* and I_2_, but not in I_0_, can be determined by calibrating I_2_ using *I*1*′*, not I_1_ (*I*2*′* is generated), and by performing stereo matching using *I*1*′* and *I*2*′*. This is because the distances to the ground for I_0_, *I*1*′* and *I*2*′* are equal. The primary challenge with this approach is the accumulation of errors. For a ground that is not flat, the true height of a plant depends on its definition. In the proposed approach, it is not easy to discover the root position of the target plant, but the average ground height can be used as a reference for measuring height, and it is believed that this is sufficient for monitoring the growth speed of the plant. In future work, these processes will be implemented and evaluated. These processes require several images and are performed at the ground base after the flight. Thus, the measurements will be performed in two steps: real-time measurement in centimeters using RTK-GNSS and measurement in millimeters on the ground base using the images captured on the previous days.

## Data Availability

The datasets generated and/or analysed during the current study are available in the https://nobuharaken.com/NatSciRep/supplementary_materials.zip repository.
